# Laparoscopic donor nephrectomy in unusual venous anatomy – donor and recepient implications

**DOI:** 10.1590/S1677-5538.IBJU.2016.0309

**Published:** 2017

**Authors:** Avinash Bapusaheb Patil, Tarun Dilip Javali, Harohalli K. Nagaraj, S. M. L. Prakash Babu, Arvind Nayak

**Affiliations:** 1 Department of Urology, M.S. Ramaiah Hospital, Bangalore

**Keywords:** Laparoscopy, Veins, Kidney Transplantation

## Abstract

**Objectives:**

Laparoscopic donor nephrectomy is now a commonly performed procedure in most of renal transplantation centers. However, the suitability of laparoscopy for donors with abnormal venous anatomy is still a subject of debate.

**Materials and methods:**

Between August 2007 and August 2014, 243 laparoscopic donor nephrectomies were performed in our institution. All donors were evaluated with preoperative three-dimensional spiral computed tomography (CT) angiography Thirteen (5.35%) donors had a left renal vein anomaly. A retrospective analysis was performed to collect donor and recipient demographics and perioperative data.

**Results:**

Four donors had a type I retroaortic vein, seven had type II retroaortic vein and a circumaortic vein was seen in three donors. The mean operative time was 114±11 minutes and mean warm ischemia time was 202±12 seconds. The mean blood loss was 52.7±18.4mL and no donor required blood transfusion. Mean recipient creatinine at the time of discharge was 1.15±0.18mg/dL, and creatinine at six months and one year follow-up was 1.12±0.13mg/dL and 1.2±0.14mg/dL, respectively. There were no significant differences in operative time, blood loss, warm ischemia time, donor hospital stay or recipient creatinine at 6 months follow-up, following laparoscopic donor nephrectomy in patients with or without left renal vein anomalies.

**Conclusion:**

Preoperative delineation of venous anatomy using CT angiography is as important as arterial anatomy. Laparoscopic donor nephrectomy is safe and feasible in patients with retroaortic or circumaortic renal vein with good recipient outcome.

## INTRODUCTION

With the advent of laparoscopic live donor nephrectomy, there has been an increase in number of donors for kidney transplantation. The first laparoscopic donor nephrectomy was performed by Ratner et al. in 1995 (1). Since then laparoscopic donor nephrectomy has become the standard of care in most transplant centers around the world. Compared with open nephrectomy, it is associated with less postoperative pain, shorter length of hospital stay, and faster return to work (2-6).

However, the suitability of laparoscopy for donors with abnormal vascular anatomy is still a subject of debate. There is sparse literature regarding the impact of left renal vein anomaly on the overall outcome of renal transplantation. The objective of this study was to describe the safety and feasibility of laparoscopic donor nephrectomy in donors with renal vein anomalies and analyze the outcome of renal transplantation in recipients of such kidneys.

## MATERIALS AND METHODS

Between August 2007 and August 2014, 243 laparoscopic donor nephrectomies were performed in our institution. A retrospective analysis was performed to collect donor and recipient demographics and perioperative data. All donors underwent standard preoperative evaluation including medical, surgical, psychological and immunological evaluation, and detailed informed consent. Three-dimensional spiral CT angiography was used to define the renal vascular anatomy and a renal isotope scan was performed to determine the choice of kidney for nephrectomy.

**Figure 1:**
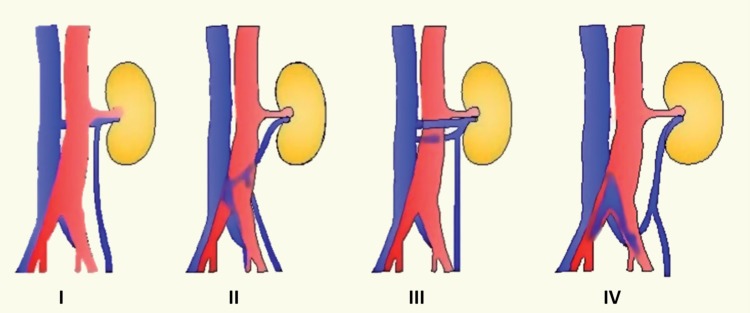
Types of left renal vein anomalies: Type I Retroaortic Left renal vein (RLRV) join the IVC in the orthotopic position, Type II RLRV join the IVC at level L4-5, Type III - circumaortic left renal vein, Type IV renal vein joins the left common iliac vein.

**Figure 2:**
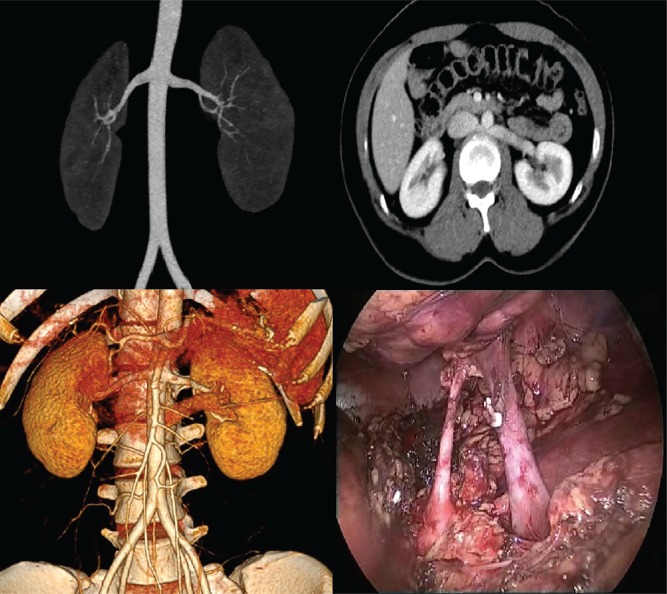
Type II retroaortic left renal vein with bilateral single renal arteries.

**Figure 3:**
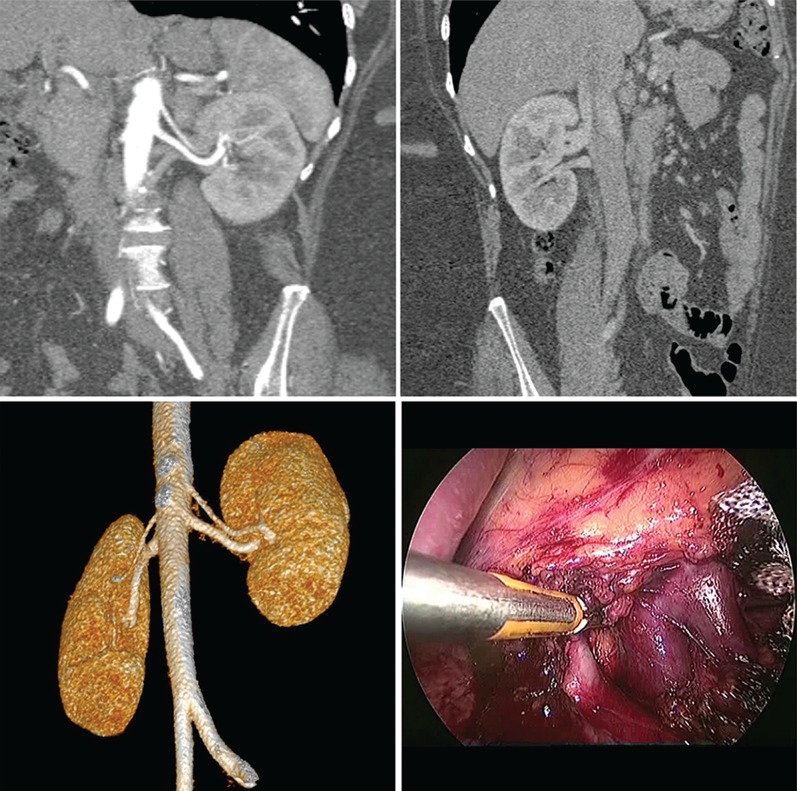
Type I retroaortic left renal vein with bilateral two renal arteries.

**Figure 4:**
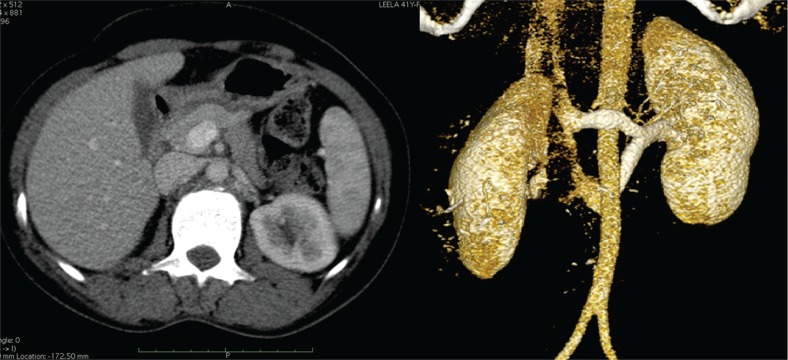
Circumaortic left renal vein.

Left renal vein abnormalities are categorized into four types (7). Types I, II and IV are “retroaortic” left renal veins, while type III is considered as “circumaortic” vein. Type I retroaortic left renal vein typically joins the IVC in the orthotopic position, while type II retroaortic left renal vein joins the IVC at level L4-5. The type IV retroaortic vein joins the left common iliac vein. The circumaortic or type III left renal vein anomaly has both a pre-aortic as well as a retroaortic component (Figure-1).Information on donor age, gender, body mass index (BMI), relation to the recipient and type of renal vein anomaly was collected. Of the 243 donors, 13 (5.35%) donors had a left renal vein anomaly. Among these, four had a type I retroaortic vein, seven had type II retroaortic vein and three donors had a circumaortic vein. Among these 13 patients with left renal vein anomalies, 11 patients had a single left renal artery (Figure-2) while one patient with type I retroaortic vein had bilateral two renal arteries (Figure-3) and one patient had bilateral two renal arteries with right two renal veins and left circumaortic vein (Figure-4). Laparoscopic right donor nephrectomy was performed in 38 patients hence they were excluded from the study. Remaining 192 patients without any left renal vein anomaly had undergone laparoscopic left donor nephrectomy. Of these 192 patients, 20 patients had multiple left renal arteries and left kidney was selected due to anomalous right renal vein/artery. Surgical data included operative time, warm ischemia time, estimated blood loss, complications, and nadir serum creatinine.

The laparoscopic procedure was performed transperitoneally. All donor nephrectomies were performed by either one of two surgeons: HKN/TDJ, and all renal transplants were performed by a single recipient team. Briefly, dissection was started at the lower pole of kidney and the ureter and gonadal vein were dissected. The gonadal vein was traced towards the left renal vein. The adrenal vein, gonadal vein and lumbar vein (if present) were identified, clipped and transected. In case of retroaortic vein, renal vein was dissected up to lateral border of aorta and clipped using two Hemolock clips (Weck® clips) and one metallic clip at the donor side. Renal artery was clipped using two Hemolock clips and one metallic clip. In two patients with circumaortic veins, smaller caliber retroaortic component was sacrificed. Kidney was delivered out through Pfannenstiel incision.

Operative time was defined as the time from the initial skin incision to delivery of the kidney to the recipient transplant team. Warm ischemia time was calculated as the time from renal artery ligation to immersion of the kidney in ice slush. Information was also collected on recipient allograft function, including serum creatinine at the time of discharge, at six months and at one year follow-up, as well as complications. Delayed graft function was defined as the patient requiring dialysis postoperatively.

Statistical analysis was performed using Instat® software (GraphPad Software, San Diego California USA). Both discrete and continuous variables were analyzed with the Student’s t-test. A P value of less than 0.05 was considered statistically significant.

## RESULTS

Donor demographic data, including age, gender, BMI, type of left renal vein anomaly, operative time, warm ischemia time and blood loss for 13 donors who underwent transperitoneal laparoscopic donor nephrectomy are listed in Table-1. There were two (15%) men and 11 (85%) women, with a mean age of 44.5±7.1 years. The mean operative time was 114±11 minutes and mean warm ischemia time was 202±12seconds.There were no intraoperative complications. The mean blood loss was 52.7±18.4mL,and no donor required blood transfusion. Donors were discharged on the third postoperative day and no donor required readmission. Mean donor creatinine preoperatively and at discharge was 1.0±0.14mg/dL and 1.27±0.18mg/dL, respectively.

**Table 1:** Donor characteristics.

Sl. No.	Donor Age (Years)	Sex	BMI (Kg/m^2^)	Type of renal vein anomaly	Number of renal arteries	Operative time (minutes)	Warm ischemia time (seconds)	Blood loss (mL)	Complications	Serum Creatinine (mg/dL)	

										Before surgery	At discharge
1	46	F	26.2	Retroaortic type II	1	118	212	45	Nil	1.1	1.3
2	30	F	22.3	Retroaortic type II	1	112	194	60	Nil	0.9	1.2
3	41	F	24.6	Retroaortic type I	1	109	190	25	Nil	1	1.2
4	52	M	27.2	Retroaortic type II	1	103	210	40	Nil	1.1	1.4
5	41	F	24.5	Circumaortic	2	133	205	85	Nil	0.9	1
6	47	F	26.8	Retroaortic type II	1	112	188	70	Nil	0.8	1.1
7	53	F	27	Retroaortic type I	1	114	217	65	Nil	1.3	1.6
8	36	M	23.4	Retroaortic type I	1	108	196	80	Nil	0.8	1
9	43	F	24.1	Retroaortic type II	1	98	208	45	Nil	1	1.3
10	39	F	25.3	Retroaortic type II	1	115	220	55	Nil	0.9	1.3
11	46	F	27.4	Circumaortic	1	124	185	40	Nil	1.1	1.4
12	54	F	28.5	Retroaortic type I	2	136	208	45	Nil	1	1.3
13	50	F	29.2	Retroaortic type II	1	103	190	30	Nil	1.1	1.5

**M=** Male; **F=** Female; **BMI =** Body mass index

Recipient outcomes are shown in Table-2. Mean recipient age was 45.2±7.3 years. The mean cold ischemia time was 50.2±8 minutes. The mean recipient blood loss was 317±39mL and mean hospital stay was 11.3±2.4 days. One patient required temporary dialysis after surgery due to delayed graft function due to acute tubular necrosis. Mean recipient creatinine at the time of discharge was 1.15±0.18mg/dL, and creatinine at six months and one year follow-up was 1.12±0.13mg/dL and 1.2±0.14mg/dL, respectively. We routinely used ureteral stents in all uretero-vesical anastomosis and these stents were removed after 21 days.

**Table 2:** Recipient outcomes.

Sl. No.	Recepient Age (years)	Sex	Cold ischemia time (minutes)	Blood loss (mL)	Hospital stay (days)	Graft related complications	Serum Creatinine (mg/dL)		

							At discharge	At 1 month	At 6 months
1	50	M	52	280	11	Nil	1.3	1.2	1.3
2	33	M	48	310	12	Nil	1	1.1	1.1
3	38	M	45	350	9	Nil	1.2	1.2	1.3
4	46	F	53	290	13	Nil	1.4	1.3	1.4
5	45	M	67	285	11	Nil	0.9	1	1
6	51	M	43	370	10	Nil	1	1	1.2
7	45	M	40	335	8	Nil	1.3	1.3	1.2
8	40	M	53	390	12	Nil	0.9	1	1.1
9	35	M	41	325	17	Delayed graft function	1.2	1	1
10	44	M	47	290	11	Nil	1.2	1.3	1.4
11	49	M	54	265	14	Nil	1.4	1.2	1.3
12	58	M	63	340	9	Nil	1	1	1.1
13	54	M	47	285	10	Nil	1.1	1	1.2

**M=** Male; **F=** Female

These results were compared to the outcomes of 192 patients without left renal vein anomaly (Table-3). Incidences of multiple left renal arteries were comparable in both the groups. There was no statistically significant difference in donor outcomes(operative time, blood loss, warm ischemia time) and recipient outcomes (serum creatinine at 6 months follow-up).

**Table 3:** Comparison of demographics and outcomes in patients with and without left renal vein anomaly.

			Donors with left renal vein anomalies (n=13)	Donors without left renal vein anomalies (n=192)	P value
Donor age (years), mean±SD			44.5±7	48.4±8.1	>0.05
Recipient age (years), mean±SD			45.2±7.3	40.1±6.9	<0.05
Multiple left renal arteries (%)			2 (15.4)	20 (10.4)	
Donor BMI (Kg/m2), mean±SD			25.9±2.0	25.5±3.7	>0.05
Operative Details	Operative time (minutes), mean±SD		114±11	109±17	>0.05
	Blood loss (mL), mean±SD		53±18	64±23	>0.05
	Warm ischemia time (seconds), mean±SD		202±12	211±18	>0.05
Hospital stay (Donors)			60 Hours	60 Hours	
Recipient outcomes	Delayed graft function (%)		1 (7.7)	11 (5.7)	
	Cold ischemia time (minutes), mean±SD		50.2±8	54.6±11	>0.05
	Serum Creatinine at 6 months (mg/dL), mean±SD		1.12±0.13	1.21±0.2	>0.05

**SD=** Standard deviation; **BMI=** Body mass index

## DISCUSSION

Incidence of end stage renal disease and number of kidneys available for renal transplant has always been a major medical concern. With the advent of laparoscopic donor nephrectomy, there has been increase in live donor pool over last decade (8). Laparoscopic donor nephrectomy offers low morbidity, shorter length of hospitalization, less pain medication requirements, and reduced convalescence as compared to open donor nephrectomy (9).

The left kidney is favored for laparoscopic nephrectomy because it provides a graft with a longer renal vein (2,3). Traditionally, right open donor nephrectomy is chosen when the left kidney has multiple renal arteries or veins or other vascular anomalies. Major concernin right-sided laparoscopic donor nephrectomy is short length right renal vein which is further shortened by use of vascular clips. There is increased risk of vasospasm and iatrogenic vascular injury during laparoscopic right donor nephrectomy as the right renal artery is located directly posterior to short right renal vein. Some authors have reported a higher potential for vascular complications with eventual graft loss with laparoscopic right donor nephrectomy (10,11).

The most common renal venous anomaly is the occurrence of dual renal veins, accounting for 15%-30%, frequently on the right side (12-15).Circumaortic and retroaortic variants constitute the most common anomalies of the left renal vein with incidence of 6.2%-14% (10,16,17).Because of the higher risk of vascular injury, the presence of a circumaortic or retroaortic renal vein has previously been considered a relative contraindication for left donor nephrectomy by some surgeons (18).Some authors have reported that there was no significant difference regarding parameters such as operative time, warm ischemia time, length of allograft vessels, and estimated blood loss in patients with circumaortic or retroaortic renal vein when compared to control group (19,20).

In this retrospective study, we analyzed safety and feasibility of laparoscopic donor nephrectomy in patients with left renal vein anomaly and we compared donor and recipient outcomes with group of patients without left renal vein anomaly. The use of CT angiography allows preoperative identification of venous anomalies (21). In our hospital, 243 patients underwent laparoscopic donor nephrectomy. On preoperative evaluation with three-dimensional spiral CT angiography, 13 patients (5.35%) were diagnosed to have left renal vein anomaly in form of retroaortic vein (11) or circumaortic vein (2). Retroaortic vein will have an abnormal course posterior to aorta. Adrenal, gonadal, and lumbar veins may enter the renal vein at abnormal position. Hence, after meticulous dissection and control of these tributaries, retroaotic vein can be clipped at the level of lateral border of aorta. In case of circumaortic vein, usually retroaortic component will have smaller caliber and it can be safely sacrificed. Preaortic component can be clipped at the opening into IVC. Careful preoperative radiological evaluation of vascular anatomy is mandatory and intraoperative potential variation in vascular anatomy has to be kept in mind. In our experience, operative time and warm ischemia time were not prolonged. The mean warm ischemia time was 202±12seconds and mean operative time was 114±18 minutes. One-year graft survival was 100%.

## CONCLUSIONS

Preoperative delineation of venous anatomy using CT angiography is as important as arterial anatomy. Laparoscopic donor nephrectomy is safe and feasible in patients with retroaortic or circumaortic renal vein with good recipient outcome.
